# The Response of the Mediterranean Gorgonian *Eunicella singularis* to Thermal Stress Is Independent of Its Nutritional Regime

**DOI:** 10.1371/journal.pone.0064370

**Published:** 2013-05-08

**Authors:** Leïla Ezzat, Pierre-Laurent Merle, Paola Furla, Alexandre Buttler, Christine Ferrier-Pagès

**Affiliations:** 1 School of Architecture, Civil and Environmental Engineering (ENAC), Ecole Polytechnique Fédérale de Lausanne (EPFL), Lausanne, Switzerland; 2 UMR 7138 UNS-UPMC-CNRS Equipe Symbiose Marine (SYMAR), Université Nice Sophia-Antipolis, Nice, France; 3 Ecophysiology Team, Centre Scientifique de Monaco, Monaco, Principality of Monaco; 4 Swiss Federal Institute for Forest, Snow and Landscape Research (WSL), Lausanne, Switzerland; 5 UMR CNRS 6249 Laboratoire de Chrono-Environnement, Université de Franche-Comté, Besançon, France; King Abdullah University of Science and Technology, Saudi Arabia

## Abstract

Over the last few decades, sessile benthic organisms from the Mediterranean Sea have suffered from the global warming of the world's oceans, and several mass mortality events were observed during warm summers. It has been hypothesized that mortality could have been due to a nutrient (food) shortage following the stratification of the water column. However, the symbiotic gorgonian *Eunicella singularis* has also presented a locally exceptional mortality, despite its autotrophic capacities through the photosynthesis of its dinoflagellate symbionts. Thus, this study has experimentally investigated the response of *E. singularis* to a thermal stress (temperature increase from 18 to 26°C), with colonies maintained more than 2 months under four nutritional diets: autotrophy only (AO), autotrophy and inorganic nitrogen addition (AN), autotrophy and heterotrophy (AH), heterotrophy only (HO). At 18°C, and contrary to many other anthozoans, supplementation of autotrophy with either inorganic nitrogen or food (heterotrophy) had no effect on the rates of respiration, photosynthesis, as well as in the chlorophyll, lipid and protein content. In the dark, heterotrophy maintained the gorgonian's metabolism, except a bleaching (loss of pigments), which did not affect the rates of photosynthesis. At 24°C, rates of respiration, and photosynthesis significantly decreased in all treatments. At 26°C, in addition to a decrease in the lipid content of all treatments, a bleaching was observed after 1 week in the AO treatment, while the AH and AN treatments resisted three weeks before bleaching. These last results suggest that, temperatures above 24°C impair the energetic reserves of this species and might explain the mortality events in the Mediterranean.

## Introduction

Gorgonians are among the most emblematic and representative organisms of the Mediterranean sublittoral communities [1]. These ecosystem engineer species play important ecological roles, not only in the plankton-benthos coupling, but also because they provide shade and shelter to numerous other species, and therefore largely contribute to the biomass and diversity of the benthic community [2]. Thus, any environmental perturbation inducing significant changes in their abundance could affect the proper functioning and organization of the Mediterranean benthic ecosystem.

Over the last few decades, gorgonians and other sessile organisms have suffered from the rapid seawater warming observed throughout the world's oceans, and showed increased events of mass mortalities and/or diseases [3]–[8], as often documented for tropical corals [9]–[11]. Thereby, after particularly warm summers, when seawater temperatures increased to and above 24°C during several weeks, the four Mediterranean gorgonian species (*Paramuricea clavata*, *Eunicella singularis*, *Eunicella cavolinii* and *Corallium rubrum*), were impacted over kilometers in the North-Occidental Mediterranean, [3], [4], [6], [12], attracting the attention of the scientific community. Several studies have therefore monitored *in situ* the growth, health and reproductive capacities of the gorgonian populations during and after a mass mortality event, to assess its impact on the structure and dynamics of the benthic community [6], [12]–[14]. Fewer studies have considered the physiological response of gorgonians to a thermal stress [15]–[17]. The latter studies showed that deep populations of *E. singularis* were more resistant to a seawater temperature increase to 24–26°C than shallow populations, and even more resistant than other species. Another study [6] linked the mortality events to a particularly strong summer stratification of the water column, and a possible reduction in food resources.

The aim of the present work was therefore to improve our knowledge on factors affecting the thermal sensitivity of *E. singularis* (Esper, 1791), one of the most impacted species [4], [12], which remained affected several years after the stress [18]. As suggested by Coma et al. [19], one important factor is the availability of food resources to sustain gorgonian metabolism during a thermal stress. In terms of energetic budget, *E. singularis* is an interesting model species because, as many other tropical scleratinian corals, it has a dual feeding mode, both through auto- and heterotrophy. Indeed, it is the only Mediterranean gorgonians to live in symbiosis with a dinoflagellate of the genus *Symbiodinium*, also commonly named zooxanthellae. The symbionts of tropical and temperate scleractinians are known to transfer most of the photosynthesized carbon (photosynthetates) to their host, which has therefore access to this autotrophic nutrition [20], [21]. Besides, like any animal *E. singularis* feeds on dissolved and particulate organic matter (heterotrophy), composed mainly of algae and zooplankton [22]–[24]. In many tropical anthozoans, heterotrophy increases the general metabolism and can sustain the whole metabolism during stress events [25].

During thermal stress, *E. singularis* may lose both its autotrophic and heterotrophic feeding capacities. Indeed, under stressful conditions, heterotrophy is generally affected by polyp retraction, which decreases prey capture [26], and by the water column stratification, which prevents the upwelling of nutrients and the subsequent development of phyto-and zooplankton. In absence of heterotrophy, most nutrients have to be supplied by symbiont photosynthesis, which is however itself affected during thermal stress [26]. Indeed, in many anthozoans, elevation of seawater temperature generally induces bleaching, characterized by the loss of symbionts and/or their associated pigments [27], with a concomitant reduction in the rate of photosynthesis and autotrophic inputs. Nevertheless, these processes are still poorly known and there is a need to disentangle both nutrition modes and their role in the corals fitness and resistance to environmental changes

In order to assess the effect of climate change on Mediterranean populations of *E. singularis*, a better understanding of the auto- and heterotrophic energy acquisition in normal and stressful conditions is required. For this purpose, we exposed several colonies to 4 trophic conditions across a range of temperatures that might be experienced *in situ* (from 18 to 26°C): autotrophy only, autotrophy supplied with inorganic nitrogen, autotrophy and heterotrophy, heterotrophy only (organisms kept in the dark). The aims of the study were to: 1) evaluate the effect of auto-and heterotrophy, in combination or alone, on the protein, chlorophyll and lipid content, as well as on the rate of photosynthesis of *E. singularis* under non-stressful conditions, and 2) determine the response of *E. singularis* to a thermal stress, when maintained under the different feeding conditions. The results obtained will allow us to gain a better knowledge of the trophic functioning of *E. singularis* under laboratory conditions, to draw inferences about what might be happening in the field. The general hypothesis tested was that nutritional mode and temperature affect the performance of *E. singularis* and that these effects can help explain the observed mass mortality and disease events in the field. Several predictions can be made: 1) under non stressful conditions, heterotrophy, in combination with autotrophy, increases the tissue reserves and eventually the rate of photosynthesis, as previously observed in many, but not all, scleractinian tropical species [25]; 2) in addition, heterotrophy only sustains the basic metabolism of *E. singularis* in the dark, and addition of inorganic nitrogen promotes photosynthesis, as for plants; 3) under stress conditions, the loss of autotrophic energy, following thermally-induced bleaching, is compensated by heterotrophy, as observed in some tropical coral species [28], [29]; 4) Alternatively, supply of inorganic nitrogen could also maintain the rates of photosynthesis. Finally, gorgonians maintained in the dark should suffer less from the thermal stress, since no reactive oxygen species, generally produced during photosynthesis [30], would induce oxidative stress in these gorgonians.

## Materials and Methods

### Biological Material

Experiments were performed on twelve mother colonies (named A to L) of the symbiotic gorgonian *Eunicella singularis* (Esper, 1791) which were randomly sampled by SCUBA diving off Sète, North West Mediterranean Sea (43°19′13.25′′N, 3°59′24.48′′E) in late January 2012. These mother colonies were located in shallow waters (∼10–15m). Samples were maintained in aerated cool water boxes and directly transferred to the laboratory. They were first acclimated at their in situ temperature of 16°C for a week, until they recovered from sampling. About 32 gorgonian tips (5 to 7 cm long) were then cut from each mother colonies (384 tips), labeled, and distributed in eight experimental tanks placed under controlled conditions at 18°C.

### Experimental Setup

The experimental setup included eight 20l tanks supplied with (oligotrophic) Mediterranean seawater pumped from 50 m depth at a flow rate of 20 l h^−1^. Tanks were grouped by two in order to have nubbins from six colonies per aquarium, or the twelve mother colonies represented in two tanks. Acclimation to laboratory conditions lasted for one week with a constant temperature of 18 ± 0.5°C. Following the acclimation week, four nutritional regimes (kept during the whole experiment) were established and maintained at a constant temperature of 18±0.5°C during two months. The four regimes (2 tanks per regime) were: autotrophy only (AO), autotrophy and ammonium enrichment (AN), heterotrophy only (HO) and heterotrophy + autotrophy (HA). AO consisted in exposing gorgonian tips to an irradiance of 125–150 µmol photons m^−2^ s^−1^, on a 12h light: 12h dark regime, to match as closely as possible the light intensity received by the gorgonians *in situ*. For AN diet, gorgonians were maintained in the same light level as in AO, but also received during half an hour, a daily pulse of 3 µM NH_4_, sampled in a mother solution of NH_4_Cl (80 mM). HO diet consisted in maintaining gorgonian tips in total darkness but feeding them, five times a week, with an equal quantity of krill, grinded frozen shrimps and mussels given at repletion. They were also fed twice a week with *Artemia salina* nauplii. For HA, gorgonian tips were exposed to an irradiance of 125–150 µmol photons m^−2^ s^−1^ and fed as for the HO diet.

After two months at 18°C, several physiological measurements (as described below) were performed and temperature was then increased in order to imitate a thermal stress event as monitored in the Mediterranean Sea [31]. It was first raised from 18°C to 22°C and kept constant during 10 to 12 days, then again from 22°C to 24°C and kept constant during 10–12 days. After this period, temperature was finally raised from 24°C to 26°C and kept constant during 3 weeks. Indeed, Rodolfo-Metalpa et al.[32] showed that during warm summers, the temperature of the surface layer increased from 20°C mid-June to 24°C mid-July, and then remained at elevated temperatures (>24°C) until August. Moreover, this thermal increase is similar to the one performed in a previous study [15] and allows comparison of the results obtained. Constant seawater temperature was maintained using temperature controllers (Toshniwal N6100, Toshcon®, West Instruments, Brighton, East Sussex, UK; ±0.1°C) and submersible resistance heaters (Visi-Therm® Deluxe, Aquarium Systems, Sarrebourg, France). Salinity values were constant at 38 PSU. All tanks were cleaned two times per week in order to prevent algal growth. Samples were collected first at the end of the 2 months period at 18°C (samples called T18), then at the end of the first week at 24°C and 26°C (samples called T24 and T26_1) and finally at the end of the third week at 26°C (samples called T26_3). Sample tips were used to assess the photosynthetic performances of the gorgonians, and then frozen at −20°C for the determination of chlorophyll, protein and lipid concentrations. At all sampling times, additional tips were directly frozen at −20°C to allow the determination of both the symbiont density and the two cellular stress marker levels.

### Measurements

#### Chlorophyll concentration

Prior to the determination of chlorophyll content of the samples used in the experiment, two different protocols of chlorophyll extraction were tested on independent gorgonian tips to obtain the best fit between chlorophyll determination and solvent toxicity [33], [34]. Tremblay et al. [34] method was based on acetone solvent extraction with a 4°C overnight incubation time, while Ritchie [33] protocol compared chlorophyll extraction according to three different solvents: acetone, methanol and ethanol with a 4°C overnight incubation time. The methanol and ethanol based protocols gave similar results while the acetone protocol was significantly less efficient in extracting chlorophyll pigments (ANOVA, p < 0.05, data not shown). We therefore chose ethanol solvent from Ritchie [33] protocol for the experiment. For this purpose, six gorgonian tips (representing six different colonies) that were first used for photosynthesis measurements, were each introduced in a glass tube containing pure ethanol. Pigments were extracted at −20°C during 24h, and this step was repeated twice to extract all pigments. The extracts were then centrifuged at 11, 000g for 10 min, and chl *a* and *c2* were measured according to Jeffrey and Humphrey [35]. Data were normalized to the tip surface area of gorgonians, which was measured using a caliper, taking into account the length and the width of the tip.

#### Protein content

The protein concentration was assessed by incubating gorgonian tips (3 tips per tank, representing six different colonies) in a water bath at 90°C for 30 min with a 1N NaOH solution. Samples were then placed overnight at 4°C. Protein standards were prepared using bovine serum albumin (BSA, Interchim) across a concentration range from 0 to 2000 μg ml^−1^ and, as for the gorgonian samples, incubated at 60°C for 30 min in 96-well microplates with a dye reagent (Uptima Reagents, Interchim). Samples were then homogenized for 30s at 400 r.p.m on a microplate shaker. Finally, absorbance was measured at 560nm and protein contents were normalized per surface area, measured as described above.

#### Lipid biomass

The lipid concentration was assessed by incubating gorgonian tips (3 tips per tank, representing six different colonies) in a water bath at 40°C during 1 hour with a solution of MeOH according to the method of Bligh and Dwyer [36]. Then, an equal volume of CHCL_2_ and H_2_O was added in each sample to obtain a bi-phasic medium. After centrifugation at 2000 rpm during 10 min, the lower phase was sampled and a second rinsing was necessary to retrieve all lipids. This lipid fraction was transferred in pre-weighted tubes, and each tube was re-weighted after 24 hours evaporation. The lipid contents were normalized to the surface area of the gorgonian.

#### Photosynthesis and respiration rates

These measurements were performed on 3 tips per tank (from 3 different colonies), and therefore 6 colonies per nutritional regime. Changes in oxygen production were monitored during 30 minutes at 0 µmol photons m^−2^ s^−1^ (respiration, R) and at the culture irradiance of 125–150 µmol photons m^−2^ s^−1^ (net photosynthesis, Pn). Measurements were repeated at the end of each temperature threshold (T18, T24, T26_1 and T26_3), at the given temperature. Tips were incubated in small glass chambers, filled with a known volume of 0.45µm filtered seawater (FSW) continuously stirred with a stirring bar, and equipped with an Unisense optode (oxygen-sensitive minisensor) connected to a computer with the Oxy-4 software (Chanel fiber-optic oxygen meter, PreSens, Regensburg, Germany). Optodes were calibrated before each experiment against nitrogen-saturated and air-saturated seawater for the 0% and 100% oxygen, respectively. Light was provided by a metal halide lamp (Philips, HPIT 400W, Distrilamp, Bossee, Indre et Loire, France). Pn and R rates were estimated by regressing oxygen data against time. At the end of the incubations, gorgonian tips were frozen for the subsequent determination of their surface area (cm^2^) and chlorophyll concentration. These two parameters were used to normalize Pn, R and gross photosynthesis (Pg  =  Pn + R) measurements. Oxygen fluxes were converted to carbon equivalents based on molar weights according to [37]: *Pc*  =  *Pg* × 12/PQ and *Rc*  =  R × (12× RQ) where *P*_C_ is the amount of carbon acquired through photosynthesis; 12 is the molecular mass of C; PQ is the photosynthetic quotient, equal to 1.1 mol O_2_: mol C [20]; *R*_C_ is the amount of carbon consumed by respiration and RQ is the respiratory quotient, equal to 0.8 mol C:mol O_2_ [20] *Pc* and *Rc* rates were used to calculate P:R  =  (Pc ×12)/(Rc ×24), considering that photosynthesis was efficient during the 12 h light period while respiration ran for 24 h.When this ratio equals to or is above 1 on a 24 h basis, this shows the autotrophic capability of an organism to self-maintenance [38].

#### Chlorophyll *a* fluorescence of photosystem II (PS II)

Measurements were performed on 3 gorgonian tips per tank, for a total of 6 different colonies per nutritional regime. A Pulse Amplitude Modulation (PAM) fluorometer [DIVING-PAM, Walz, Germany, [39]] was used to assess the maximal photosynthetic efficiency of photosystems II. The minimal (F_0_) and maximal (F_m_) fluorescence yields were measured, after putting the gorgonians 5 min in the dark, by applying a weak pulsed red light (max. intensity <1 mol photon m^−2^ s^−1^, width 3µs, frequency 0.6kHz) and a saturating pulse of actinic light (max. intensity 5000 µmol photon m^−2^ s^−1^, width 800ms) on gorgonian tips by mean of an optical fiber placed at a fixed distance from tips surface area. The following equation allows calculating the maximum photosynthetic efficiency: F_v_/F_m_  =  (F_m_–F_0_)/F_m_, where F_v_ is the variable fluorescence. Rapid light curve (RLC) function of the PAM was also used to estimate at the end of each temperature threshold (T18, T24, T26_1, T26_3), the effective quantum yield (ΔF/F_m'_), after exposure for 10s to 9 different light intensities (from 0 to 2983 µmol photons m^−2^ s^−1^). The last light level was followed by the 10min dark relaxation period where ΔF/F_m'_ was calculated after 30s, 1, 3, 5 and 10 mn.

#### Symbiont density and cellular stress markers

At all sampling times, 3 additional gorgonian tips per tank, for a total of 6 different colonies per nutritional regime, were directly frozen at −20°C, without any other manipulation, to avoid stress-on stress bias. Subsequently, each of these frozen fragments was weighed, measured in size, grinded in liquid nitrogen and powdered in a mortar. The symbiont density was first measured (using a modified Neubauer hemocytometer) and cytosoluble protein extracted by tissue homogenization using short sonication cycles following the protocols previously described [14]. On crude extracts, we then measured: the total oxyradical scavenging capacity (TOSC, using a spectro-fluorometer, SAFAS, Monaco) and the degree of protein ubiquitination (using dot blots, with a rabbit anti-ubiquitin antibody, DakoCytomation). TOSC and Ubiquitination results were normalized to the amount of extracted cytoplasmic proteins (BioRad Assay Kit, using Bovine Serum Albumin as standard).

### Statistical Data Analysis

Statistical analyses were performed using Statistica 11 (Statsoft). Data were collected and tested at the end of each temperature threshold. All data were expressed as means ± standard error. Normality and homoscedasticity of the data residuals were tested using Kolmogorov-Smirnov (using Lilliefors corrections) and Levene tests, and data were log-transformed when required. A general linear model for parametric repeated measure analysis of variance (ANOVA) was performed on all response variables using the different temperature steps as dependent variables and nutritional diet and colony as categorical predictors. The assumption of sphericity (independency of the repeated measures) was tested. When not fulfilled, the hypotheses were tested using the multivariate approach (Wilks test) for repeated measurements. When there were significant differences, the analyses were followed by *a posteriori* testing (Tukey's test). P-values were considered for p<α, α = 0.05.

### Ethics Statement

Twelve mother colonies of *Eunicella singularis* (Cnidarian) were sampled off Sète, North West Mediterranean Sea (43°19′13.25′′N, 3°59′24.48′′E) in late January 2012 at 15m depth under collection permit of Sète Marine Station (OF4500055960), of the University of Montpellier II. As scientific organization, represented by Dr. François Bonhomme, Sète Marine Station is empowered to conduct sampling in nature.

## Results

### Chlorophyll, Protein and Lipid Extractions

**Figure 1:**
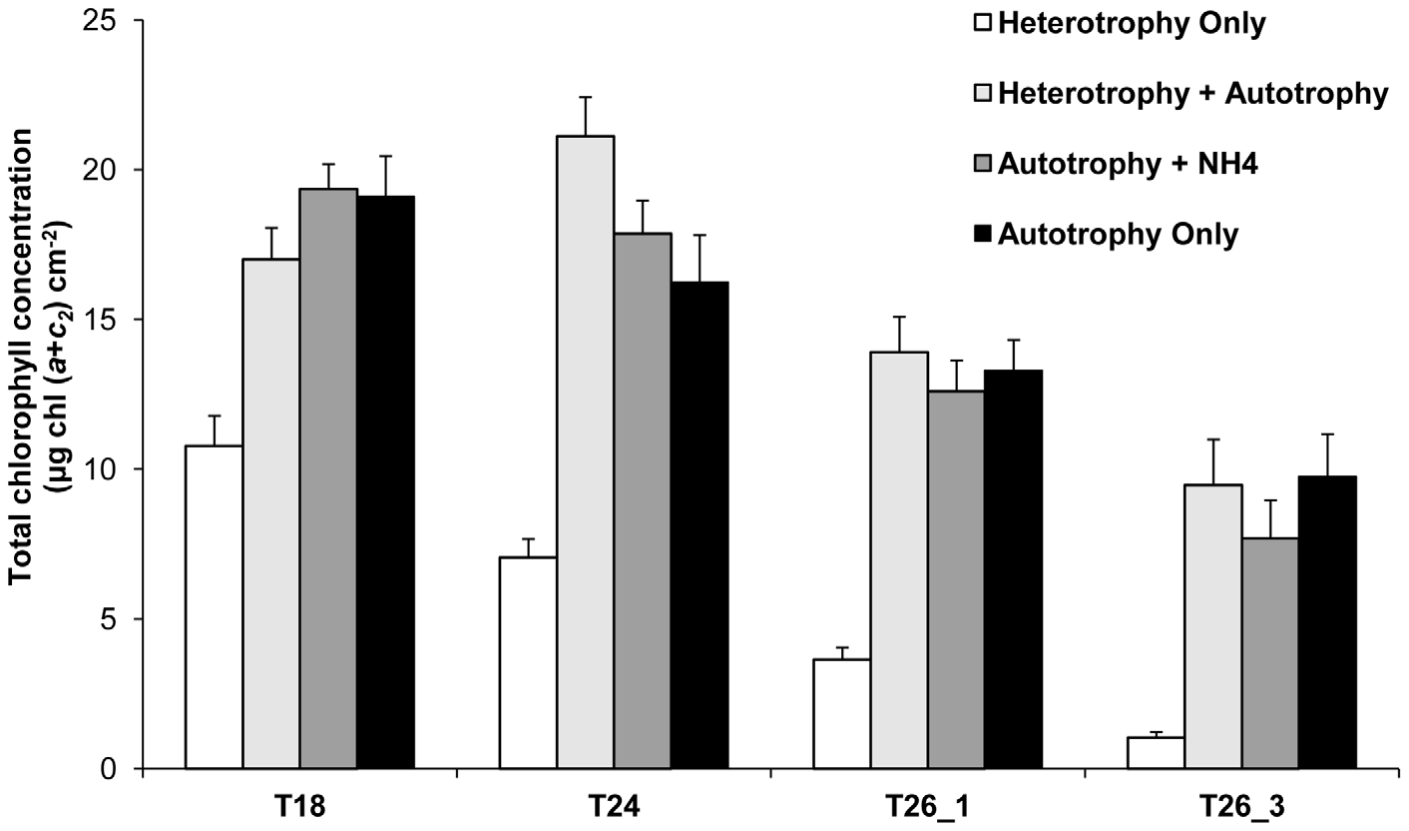
Effect of nutritional diets and thermal stress on total chlorophyll concentration of *E.singularis*. Total chlorophyll concentration (μg chl (*a*+*c2*) cm^−2^) for the different nutritional treatments according to seawater temperature (18°C, 24°C, and 26°C after 1 and 3 weeks).

There was a significant interaction between the nutritional diet and the temperature on the gorgonian chlorophyll content (Figure 1, Table 1). Indeed, HO samples showed a significant decrease of this variable as soon as temperature reached 24°C (Tukey HSD, p<0.02), and contained almost no chlorophyll in their tissue after three weeks at 26°C (Figure 1). Conversely, chlorophyll content was significantly decreased only after a week at 26°C for AO samples (Tukey HSD, p = 0.04) and after three weeks at 26°C (T26_3) for HA and AN samples (Tukey HSD, p<0.002). The different regimes and the heat stress duration exerted similar effects on symbiont density, than those observed on the chlorophyll content (data not shown). Therefore, the amount of total chlorophyll content per symbiont remained stable at 4.7±0.8 pg cell^−1^ for all regimes and stress times (except for HO-T26_3, for which the calculation was not possible due to the lack of accurate algal density determination).

**Table 1:** Results of the statistical analyses on the effects of the nutritional diet and temperature factors on the tissue parameters of gorgonians maintained in the different nutritional treatments according to seawater temperatures (18°C, 24°C, 26°C after 1 and 3 weeks).

	Degrees of freedom	P-value	F-Value
***Chlorophyll content*** (μg chl (a+c2) cm^−2^)			
Temperature	3	**P<0.00001**	119.3119
Diet	3	**P<0.00001**	94.6450
Colony	5	**0.002**	3.5740
Temperature * Diet	9	**P<0.00001**	7.5778
***Protein content*** (mg cm^−2^)			
Temperature	3	**P<0.0001**	8.8645
Diet	3	0.2315	1.5982
Colony	5	0.3408	1.2358
Temperature * Diet	9	0.2967	1.2380
***Lipid content*** (mg cm^−2^)			
Temperature	2	**P<0.0001**	13.6454
Diet	3	0.1870	1.8185
Colony	5	0.5744	0.7877
Temperature * Diet	6	0.1803	1.6047
***Ubiquitine content*** (arbitrary units)			
Temperature	3	**P<0.00001**	20.8791
Diet	3	0.7465	0.4124
Colony	5	**0.0051**	5.3431
Temperature * Diet	9	0.7720	4.0446

**Figure 2:**
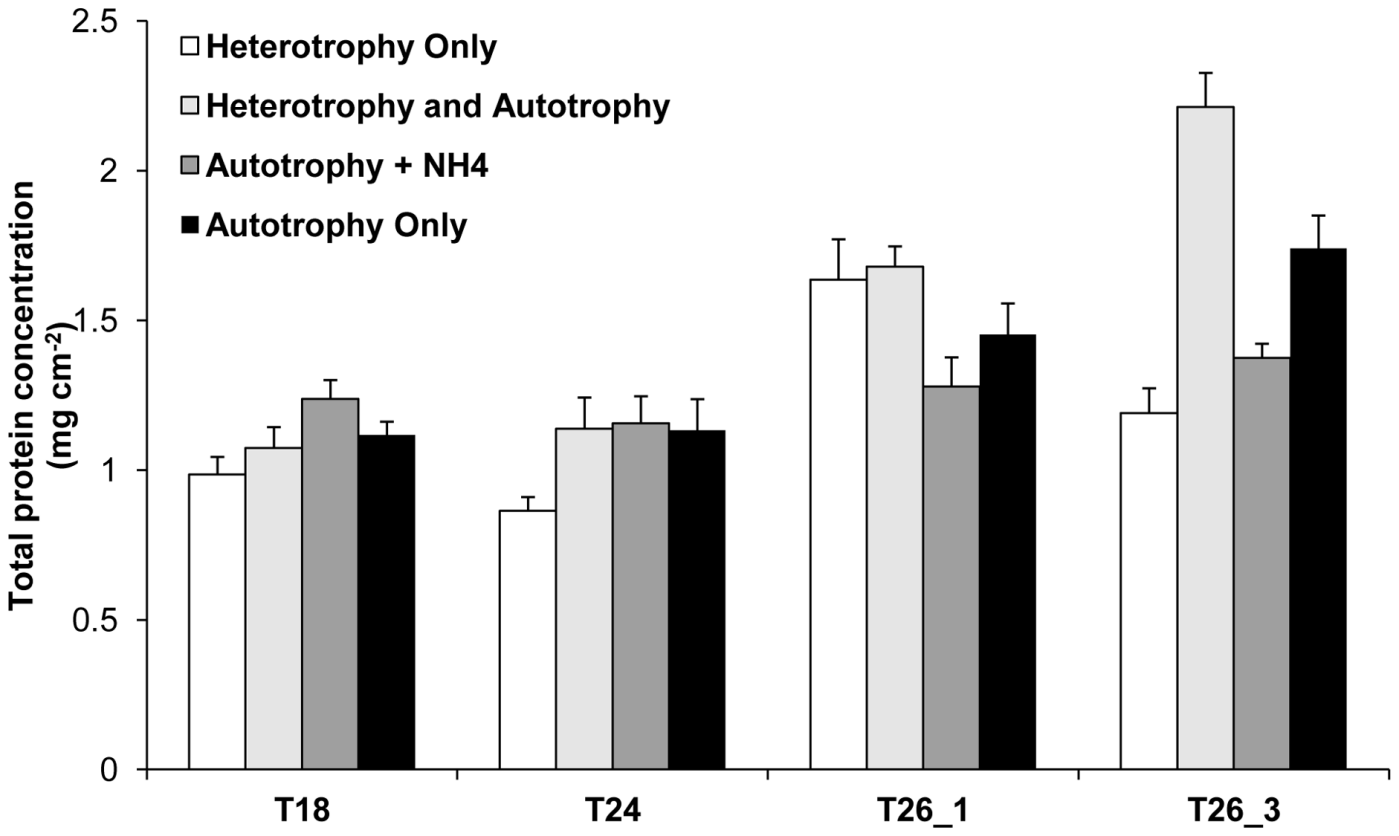
Effect of nutritional diets and thermal stress on total protein concentration of *E.singularis*. Total protein concentration (mg cm^−2^) for the different nutritional treatments according to seawater temperature (18°C, 24°C, and 26°C after 1 and 3 weeks).

The nutritional diet induced no significant difference in protein contents (0.8–1.2 mg cm^−2^) (Figure 2, Table 1). Indeed, heterotrophy allowed HO gorgonians to maintain their protein concentration at the same level than gorgonians exposed to light for the two first temperature thresholds. High temperatures had a significant effect on the protein concentration, which increased in treatments at T26_3 (Figure 2, Tukey HSD, p < 0.001) with a greater tendency for the heterotrophic diets (Tukey HSD, p> 0.05).

**Figure 3:**
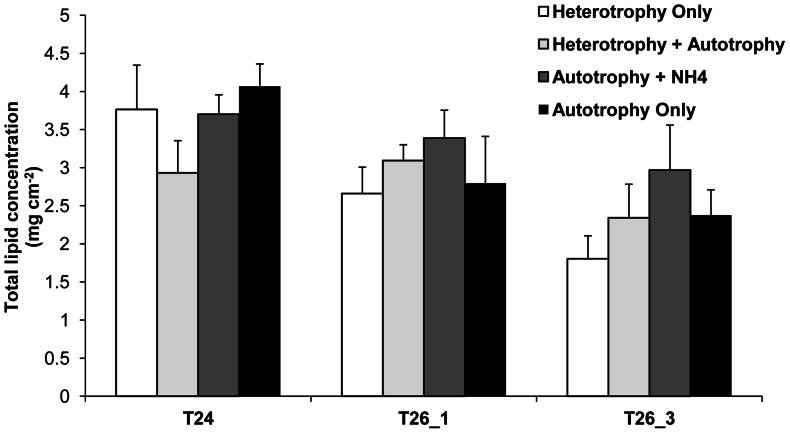
Effect of nutritional diets and thermal stress on total lipid concentration of *E.singularis*. Total lipid concentration (mg cm^−2^) for the different nutritional treatments according to seawater temperature (24°C, and 26°C after 1 and 3 weeks).

Regarding the lipid extraction, half of the 18°C samples (AO and AH) have been unfortunately lost (due to freezing problems). However, the two remaining samples were not significantly different from T24 (ANOVA, p>0.05, data not shown). We will therefore present only the results of the three following temperature steps (T24, T26_1 and T26_3). In the present case, only thermal stress had a significant effect (Table 2) by decreasing the lipid contents of all gorgonians between T24 and T26_1 (Figure 3, Tukey HSD, p<0.04).

**Table 2:** Results of the statistical analyses on the effects of the nutritional diet and temperature factors on the photosynthetic parameters of gorgonians maintained in the different nutritional treatments according to seawater temperatures (18°C, 24°C, 26°C after 1 and 3 weeks).

	Degrees of freedom	P-Value	F-Value
***Maximum quantum yield*** (Fv/Fm)			
Temperature	3	**P<0.00001**	72.1850
Diet	3	**P<0.00001**	317.8420
Colony	5	**0.0245**	5.1110
Temperature * Diet	9	**P<0.0001**	6.1240
***Effective quantum yield*** (Fv'/Fm')			
Temperature	3	**P<0.00001**	81.8900
Diet	3	**0.0157**	6.0000
Colony	5	0.8952	0.2000
Temperature * Diet	9	**P<0.00001**	8.8200
***Respiration*** (μmol O2 h^−1^ cm^−2^)			
Temperature	3	**P<0.00001**	131.3409
Diet	3	0.9687	0.0821
Colony	5	0.8652	0.3640
Temperature * Diet	9	**0.0085**	2.9039
***Net photosynthesis*** (μmol O2 h^−1^ cm^−2^)			
Temperature	3	**P<0.00001**	90.9526
Diet	3	**P<0.00001**	22.0642
Colony	5	0.2627	1.4524
Temperature * Diet	9	**0.0138**	2.8535
***Gross photosynthesis*** (μmol O2 h^−1^ cm^−2^)			
Temperature	3	**P<0.00001**	96.3068
Diet	3	0.0932	2.6025
Colony	5	0.4530	0.9999
Temperature * Diet	9	0.9058	0.5283

### Photosynthesis and Respiration Rates

In the following results, data are expressed per surface area of gorgonian colony, when not specified, but normalization per chlorophyll content is also discussed. Normalization per surface area allows comparison between treatments considering the total amount of carbon produced and respired by the gorgonian colony, while the normalization per chlorophyll content is rather an index of chlorophyll efficiency.

**Figure 4:**
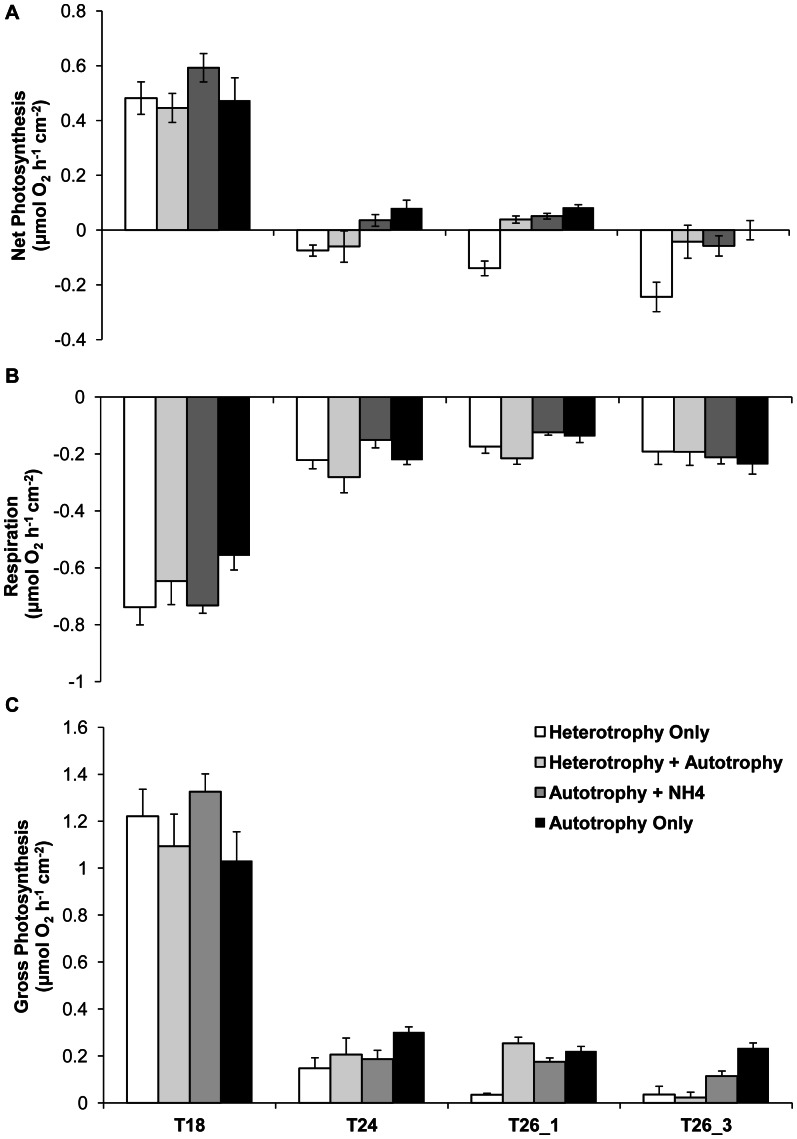
Effect of nutritional diets and thermal stress on photosynthesis and respirations rates of *E.singularis*. Net photosynthesis (Pn) (A), respiration (R) (B) and gross photosynthesis (Pg) (C) rates (μmol O_2_ h^−1^ cm^−2^) for the four nutritional treatments according to seawater temperature (18°C, 24°C, and 26°C after 1 and 3 weeks).

There was a significant interaction between the nutritional diet and the temperature for the response of R and Pn (Table 2) normalized to the surface area, but the interaction was mainly due to the HO treatment at 26°C that behaved differently than the others. Indeed, after two months at the control temperature of 18°C, no significant differences appeared between the four nutritional regimes for net photosynthesis, Pn, (Figure 4A, Tukey HSD, p>0.05) respiration rates, R (Figure 4B, Tukey HSD, p>0.05) and gross photosynthesis, Pg (Figure 4C, Tukey HSD, p>0.05). Gorgonians produced per surface area as much O_2_ as they consumed (Pn: 0.45–0.65 μmol O_2_ h^−1^ cm^−2^ and R: 0.5–0.8 μmol O_2_ h^−1^ cm^−2^). When normalized to the chlorophyll content, all treatments maintained in the light (AO, AN, AH) also presented equivalent rates of Pn (0.012±0.002 µmol O_2_ (µg chl a) ^−1^ h^−1^), and R (−0.015±0.005 µmol O_2_ (µg chl a) ^−1^ h^−1^). However, HO gorgonians had higher Pn (0.022±0.003 µmol O_2_ (µg chl a) ^−1^ h^−1^), and R (−0.034±0.005 µmol O_2_ (µg chl a) ^−1^ h^−1^), because their chlorophyll content was significantly lower. Finally, all treatments maintained at this control temperature showed a P:R ratio above 1, with a higher value for the AO treatment and a lower for the HO (Table 3).

**Table 3:** P:R (Photosynthesis-respiration ratio) of the gorgonians maintained in the different nutritional treatments according to seawater temperatures (18°C, 24°C, 26°C after 1 and 3 weeks).

Nutritional treatment	T18	T24	T26_1	T26_3
**HO: Heterotrophy Only**	1.13± 0.03	0.51± 0.04	0.19± 0.06	0.41± 0.20
**HA: Heterotrophy + Autotrophy**	1.18± 0.07	0.57± 0.11	0.82± 0.05	0.71± 0.07
**AN: Autotrophy + NH_4_**	1.24± 0.03	0.95± 0.16	0.97± 0.06	0.38± 0.07
**AO: Autotrophy Only**	1.26± 0.07	0.96± 0.10	1.16± 0.09	0.80± 0.08

After a week exposure at 24°C, Pn, R and Pg, either normalized to the surface area (Figure 4A, B, C) or to chlorophyll content, significantly and drastically decreased for all regimes compared to the control temperature of 18°C (Tukey HSD, p<0.0001), with no difference in R, Pn and Pg between nutritional treatments (Tukey HSD, p>0.05). Rates normalized to the chlorophyll content were equal to: −0.006±0.005 µmol O_2_ (µg chl a)^−1^ h^−1^) for Pn, and −0.009±0.005 µmol O_2_ (µg chl a) ^−1^ h^−1^) for R. P:R ratios decreased to below 1 in all treatments, but more drastically in the gorgonians maintained under heterotrophy (HO and HA) than under autotrophy (AO, AN) (Table 3).

After one to three weeks at 26°C, Pn, R and thus Pg normalized to the surface area remained comparable to the measurements performed at 24°C (Tukey HSD, p>0.05 respectively for T26_1 and T26_3). However, Pn of gorgonians maintained in the dark (HO) were significantly lower than measurements performed at T24 (Tukey HSD, p < 0.001). After a week at 26°C, P:R ratios of AN and AO regimes remained above or equal to 1, allowing gorgonian nubbins to still meet their metabolic needs (Table 3). However, after three weeks at 26°C, all P:R ratio decreased to below 1, indicating that autotrophic contribution no longer allowed to meet metabolic needs (Table 3).

### Photosynthetic Efficiency of Photosystem II (PSII)

**Figure 5:**
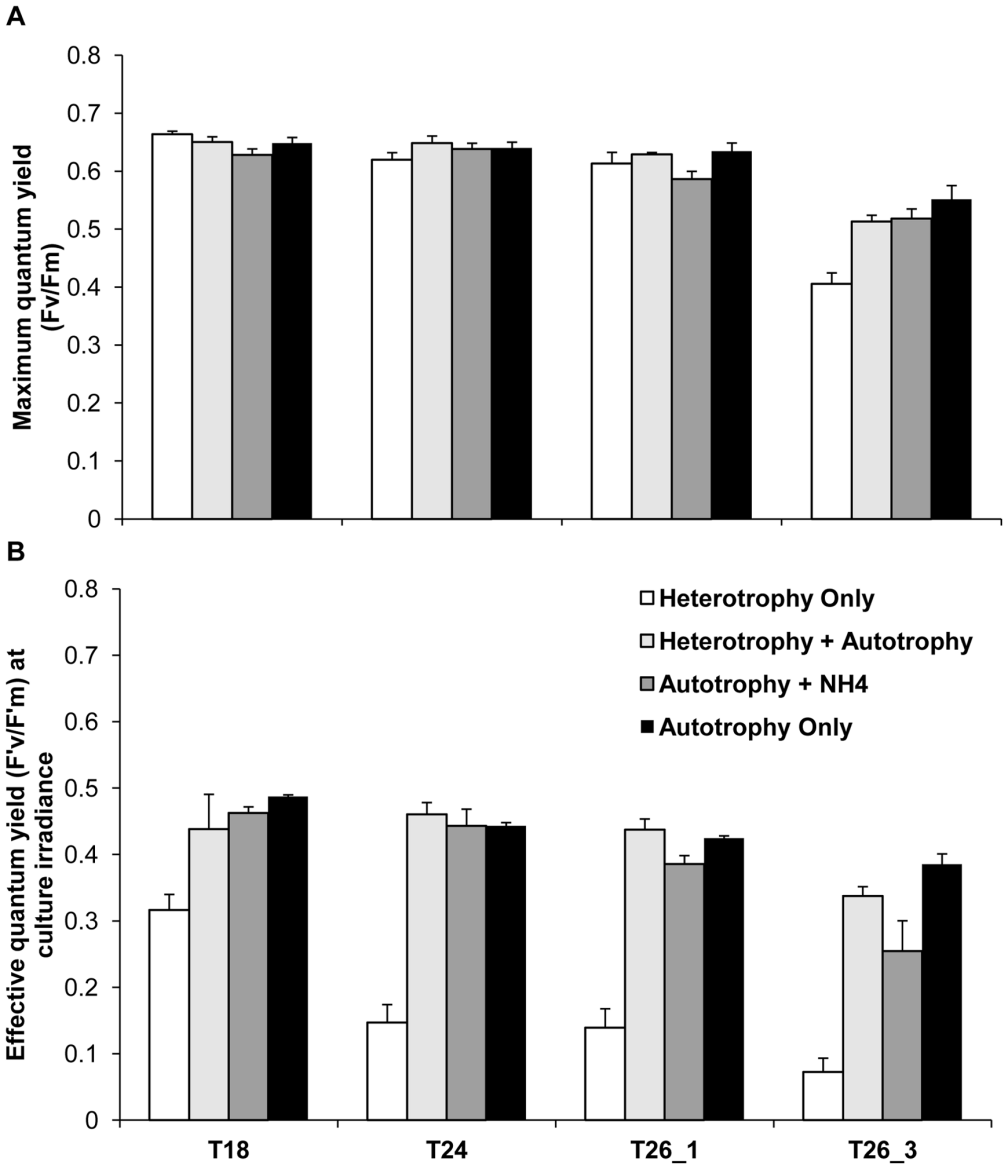
Effect of nutrional diets and thermal stress on maximum and effective quantum yield of *E.singularis*. Maximum quantum yield (F_v_/F_m_) (A), Effective quantum yield at culture irradiance (F'_v_/F'_m_) (B) for the different nutritional treatments according to sea seawater temperature (18°C, 24°C, and 26°C after 1 and 3 weeks).

**Figure 6:**
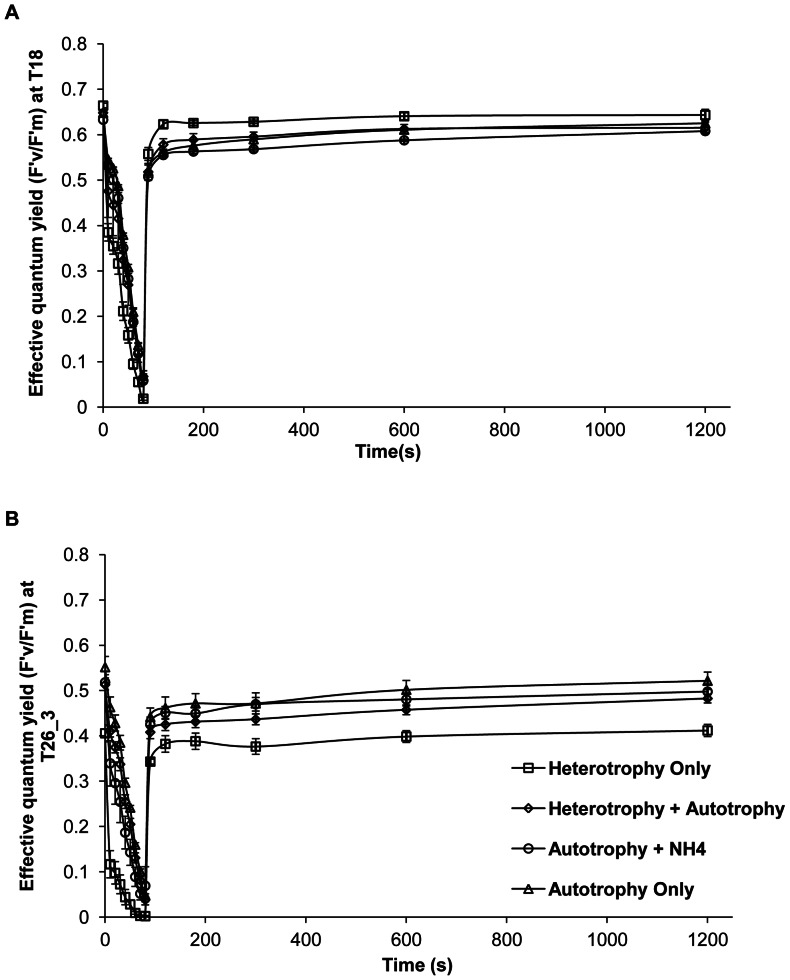
Effect of nutritional diets and thermal stress on effective quantum yield and recovery during the RLC (rapid light curve). Effective quantum yield (F'_v_/F'_m_) and recovery versus Time(s) at T18 (A) and T26_3 (B).

Overall, temperature decreased the F_v_/F_m_ in all treatments, from 0.65±0.02 at T18 and T24, to 0.4±0.15 at T26_3 (Figure 5A, Tukey HSD, p<0.05). Also, at each temperature step, maintaining the gorgonians in the dark (HO treatment) significantly lowered the F'_v_/F'_m_ compared to the other treatments (Tukey HSD, p<0.001). This lower effective photosynthetic efficiency suggests the loss of some photoprotective pigments. This is explained by a higher increase in minimal fluorescence (F_0_) for the HO treatment, compared to the others, suggesting a faster increase in inactive PSII reaction centers. There was also a significant interaction of the nutritional diet and the temperature (Table 2) on both (F_v_/F_m_) and (F'_v_/F'_m_) responses. This interaction was mainly due to a significantly lower F'_v_/F'_m_ at temperatures ≥ 24°C and lower F_v_/F_m_ at T26_3 for gorgonians maintained in the dark than for those maintained in the light (Figure 5A, B, Tukey HSD, p < 0.001). These gorgonians did not recover their initial yield, even after more than 10 minutes recovery in the dark (Figure 6). Moreover, for gorgonians maintained in the light, HA treatment had a significantly lower F_v_/F_m_ than AO treatment at T26_3 (Tukey HSD, p = 0.036) and AN treatment also showed a significant decrease in the F'_v_/F'_m_ for temperatures ≥ 26°C (Tukey HSD, p < 0.02). HA and AO treatments maintained a similar F'_v_/F'_m_ value between T18 until T26_3 (Figure 5B, Tukey HSD, p < 0.01).

### Stress Markers

**Figure 7:**
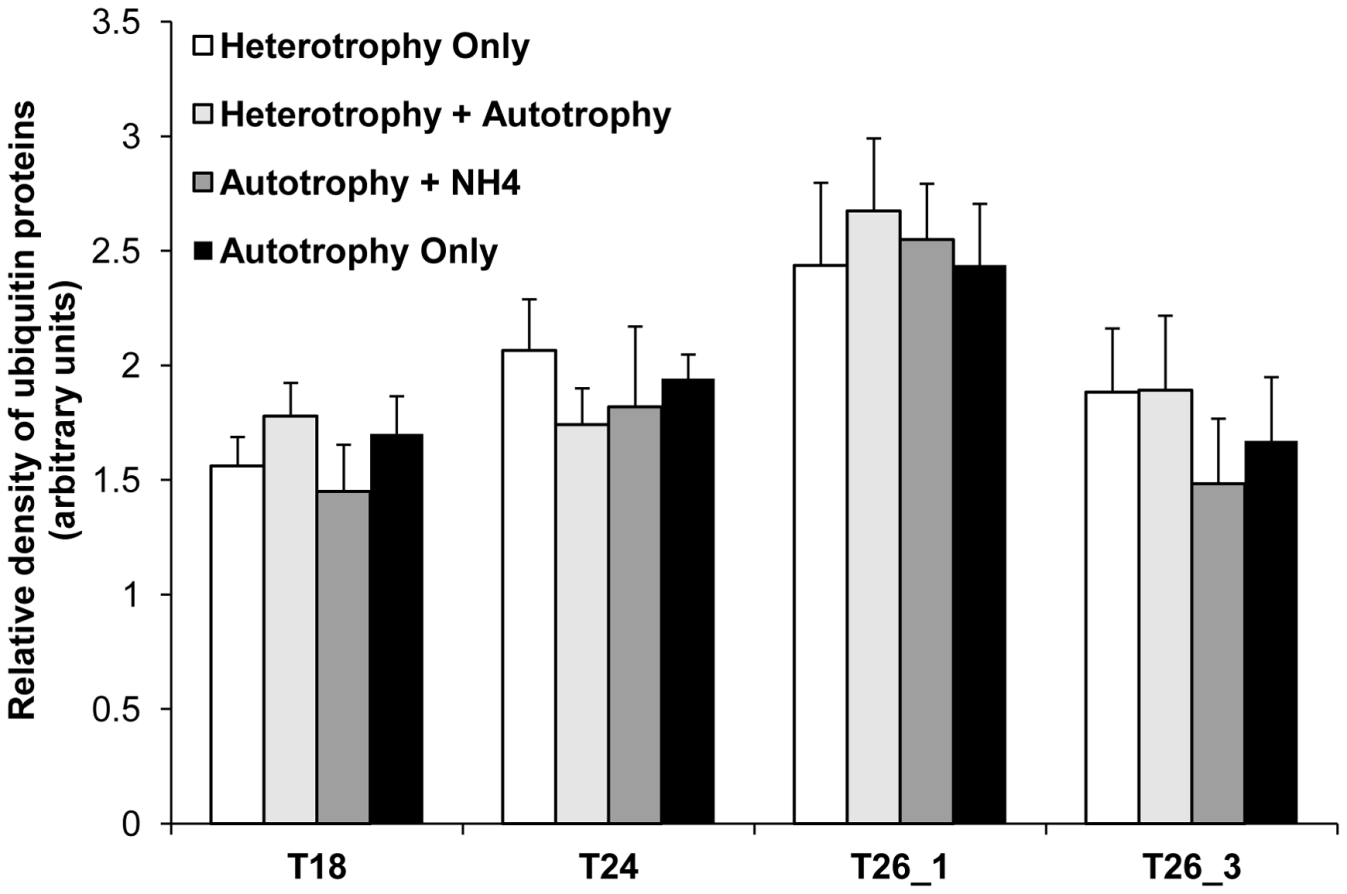
Effect of nutritional diets and thermal stress on ubiquitinylated proteins of *E.singularis*. Relative density of ubiquitin proteins (arbitrary units) for the different nutritional treatments according to seawater temperature (18°C, 24°C, and 26°C after 1 and 3 weeks).

No significant effect of the nutritional regimes and the heat stress duration was detected on the TOSC levels (ANOVA, p>0.05, data not shown), mainly due to high inter-individual variations (see the colony effects in Table 1 and 2). Measurements of ubiquitin conjugates showed a transient increase of this parameter at T26_1 (Figure 7, Table 1, Tukey HSD, p<0.0001), which returned to basal levels at the last sampling point (T26_3) (Tukey HSD, p<0.001).

## Discussion

### Nutritional Effects on *Eunicella singularis* Physiology

This paper presents for the first time the effect of autotrophy and/or heterotrophy on the metabolism and photosynthetic capacities of *E. singularis*, one of the most threatened gorgonians of the Mediterranean benthic ecosystems. This effect was evaluated on healthy and thermally-stress gorgonians. Results showed that *E. singularis* had a certain nutritional plasticity under non-stressful culture conditions. This species was indeed able to use either heterotrophy, or autotrophy only, to sustain its basic metabolism. The combination of the two nutritional regimes did not offer an additional advantage compared to the other conditions. The photosynthetic capacities of this gorgonian however rapidly collapsed as soon as temperature reached 24°C, independently of the trophic regime, and the supply of external nutrients was not used to sustain the rates of photosynthesis.

Under non-stressful conditions (18°), and in organisms maintained in the light, there was no significant difference in chlorophyll and protein contents, respiration, photosynthesis and photosynthetic efficiency, whether gorgonians were maintained in pure autotrophy, with a supply of inorganic nitrogen or with organic feeding. The lack of feeding effect on gorgonian metabolism is different from the effect generally observed on other symbiotic anthozoan species, such as on tropical [25] and temperate scleractinian corals [21], [40]–[42], for which feeding increases protein and chlorophyll concentrations, as well as symbiont densities, calcification and photosynthesis rates. It has however to be noticed that the effect of feeding on other temperate anthozoans is temperature and/or light dependent. Indeed, in the scleractinian coral *Cladocora caespitosa*, an enhancement of symbiont density by feeding was only observed under low temperatures (10–12°C, [42]) and increase in tissue biomass was significant only under high light intensity (250 µmol photons m^−2^ s^−1^, [21]). In another temperate coral species, *Plesiastrea versipora*, the only significant effect of feeding also occurred under low temperature conditions [43]. In view of these observations, the effect of feeding on the metabolism of temperate species seems to be strongly correlated with or dependent on light and temperature. This might explain why, in our experimental conditions, where light and temperature were kept at a “medium” level, feeding had no significant effect on *E. singularis* metabolism. It can also be argued that *E. singularis* did not feed on the type of prey and dissolved material given as heterotrophic diet in this experiment. However two observations proved the contrary. Gorgonians maintained in the dark for more than two months did not decrease their respiration rates and tissue biomass compared to the other treatments, indicating that, in this condition, food served as a metabolic fuel to sustain metabolism. In addition, at 24°C, fed treatments (HO and HA) presented a P:R ratio less than 1 and lower compared to the unfed treatments. Thereby, gorgonians supplied with sufficient nutrients, decreased their production of symbiont photosynthates, suggesting a “down-regulation” phenomena with a shift from auto-to heterotrophy. Heterotrophy might therefore play an important role in gorgonians living in deep waters, where light levels are generally very low. Finally, *E. singularis* photosynthesis was not enhanced following an inorganic nitrogen enrichment. In tropical corals, such enrichment induced an increase in symbiont density [44], [45], leading in some occasions to an enhancement in the rate of photosynthesis [46], but not always [45]. This lack of effect at least suggests that *Eunicella singularis* is not nutrient limited under our experimental conditions. Additional experiments using C:N measurements should be carried out to get a better understanding of the effect of heterotrophy on this species.

At 18°C, only gorgonians maintained fed in the dark, showed some physiological differences compared to the other treatments. As already discussed above, heterotrophy alone was sufficient to sustain the basic metabolism (tissue biomass and ,respiration rates), as already observed with the Mediterranean scleractinian coral *Cladocora caespitosa,* whose metabolism in the dark was sustained through heterotrophy for 2 months [21]. A bleaching however occurred in the gorgonians during this experiment, and has to be related to the occurrence, in situ, at 100 m depth, of aposymbiotic colonies of *E. singularis*. All together, these observations suggest that the loss of symbionts in the dark is a common process in this gorgonian species [47]. Indeed, such bleaching cannot be attributed to a degradation of the chlorophyll pigments in the dark, since the maximal photosynthetic efficiency (F_v_/F_m_), and the rates of photosynthesis were kept at the same level as in the light. Photosystems were rather inactivated than destructed. Symbiont loss seems thus to be due to elevated energetic costs for keeping a large symbiont population in the gorgonian tissue. This observation provides useful information about the ecology of this gorgonian species, which spends the winter months at very low levels of irradiance. It shows that temporary shading, although decreasing pigment and/or symbiont concentrations, does not have a serious impact on the autotrophic capacities of the gorgonian. Overall, symbionts of *E. singularis* behave like many phytoplankton species [48]–[50], but also like the symbionts of the Mediterranean scleractinian coral *Cladocora caespitosa* [21], which are also known to cope with a period of darkness without losing their photosynthetic capacities. For such Mediterranean symbiotic organisms, the maintenance of the photosynthetic apparatus is therefore vital for an efficient photosynthesis and an efficient input of autotrophic carbon, on return to favorable light levels.

### Effects of Thermal Stress

This experiment has shown that as soon as the seawater temperature was increased to 24°C, many physiological processes in *E. singularis*, such as the rates of respiration and photosynthesis collapsed, independently of the trophic status of the gorgonians. A similar decrease in respiration and polyp activity above 20°C has been previously observed in *E. singularis* either in laboratory [17] or *in situ*, during the summer period when seawater temperature reached 22 to 24°C [51]. Several authors [51]–[53] related this decreased metabolic activity to the “summer dormancy”, which corresponds to an adaptation to nutrient shortage conditions after the stratification of the upper water column. Gorgonians therefore tend to present a low polyp activity and a high consumption rate of internal lipid and protein reserves due to a lack of food in the surrounding environment [52]. However, results obtained in this experiment, in which half of the treatments were fed at repletion, suggest that the decrease in the general gorgonian metabolism is not only due to food shortage but rather to a real thermal stress. Indeed, both heterotrophically-fed and unfed gorgonians in this study presented the same decline in the rates of respiration, photosynthesis, and symbiont density. This lack of feeding effect on the physiology of *E.singularis* is therefore contrary to most of the previous observations made on other tropical and Mediterranean anthozoans, for which feeding generally enhances the metabolism and sustains it during thermal stress [25], [26], [29]. Another theory that has been put forward to explain the sensitivity of organisms to thermal stress is the “Oxygen- and Capacity-Limited Thermal Tolerance” [54]. It is how ambient oxygen levels shape and limit animal life. In the case of gorgonians, abnormal increase in seawater temperature may induce a stratification of the water column, reduce the amount of oxygen available, and thereby induce organisms' hypoxia. Although this process might occur in situ, it can not explain the decrease in metabolic activity observed in this study, as the aquaria were well-mixed and the seawater renewal rate was important. 

The significant and drastic decrease in oxygen production, in all treatments at 24°C, suggests that the autotrophic capacity of the gorgonians was impacted at this temperature, and reached a critical state at 26°C. This photosynthetic shut-down was not due to a photoinhibition of the photosystems, or to a degradation of the D1 protein [55], since chlorophyll concentrations, and the maximal and effective quantum yields remained at a high level during the whole experiments, only decreasing at 26°C, as observed in a previous study [15]. Gorgonians also recovered a maximal yield after a dark recovery period, suggesting that the symbionts were still photosynthetically competent. This response is also different than those observed in tropical corals, for which a decrease in oxygen production is often related to a loss in photosynthetic pigments and photosynthetic efficiency [56]–[60]. The uncoupling between chlorophyll fluorescence response and O_2_ evolution has already been observed in phytoplankton, and might have several origins. Mitochondrial respiration can lead to an underestimation of the actual oxygen production [61]. Processes involved in chlororespiration, in the Mehler reaction or in plastoquinol oxidase (PTOX) can also explain this discrepancy [62]. Indeed, the PTOX pathway, for example, is used by prokaryotic and eukaryotic cells to generate ATP without producing reductant, leading to a dissipation of light energy with reduced O_2_ production. The ATP generated is however used to support metabolic pathways, which can be the case here in the gorgonians exposed to a thermal stress. Further experiments are needed to draw the complete activation pathways involved in the heat-induced bleaching of this temperate symbiotic Cnidarian. However, since the amount of chlorophyll per cell did not change during the heat stress, the observed bleaching was associated with the degradation or the expulsion of the symbionts. Corroborating our previous observations with this same species, the level of ubiquitine conjugate remained stable at 24°C and only increased at the 26°C step, suggesting higher protein degradation processes at this stage [14]. Obviously, in the absence of proteasome inhibitors (in such circulating seawater systems), the amount of protein ubiquitine conjugates punctually reflects the very dynamic equilibriums between protein synthesis, stabilization and degradation. The fact that the ubiquitination levels returned to basal for a longer hyperthermia exposure (T26_3) is coherent with the fact that no tissue necrosis was observed throughout the experiment and is also reminiscent with the observations made in a previous experiment [15]. In this experiment [15], there was an effect of the thermal history on the gorgonian capacity to resist thermal stress. Indeed, calcification rates and photosynthetic efficiency were maintained at a higher level in gorgonian colonies collected in deep (35m) than in surface waters (ca. 15m). The main hypotheses were that either deep colonies had access to a larger quantity of zooplankton and particulate matter that supplied energy to resist to the stress, or that these deep colonies were exposed less frequently to high temperature levels and oxidative stress. It is obvious, from this study, that a lack of food cannot explain the lower resistance of surface populations to thermal stress, their sensitivity might therefore be due either to a higher occurrence of high temperature events, or to a combination of high temperature and high irradiance levels, inducing oxidative stress.

### Conclusion

Under non-stressful conditions (18°C, 150 µmol photons m^−2^ s^−1^), this study has shown that *E. singularis* was mainly autotroph, since heterotrophy, in our experimental conditions did not change any of the parameters tested. This is contrary to what was observed in many other scleractinian tropical species for which heterotrophy enhanced their metabolism, independently of the irradiance received. In the dark, however, feeding alone was able to maintain gorgonian's metabolism. This has important implications for understanding the ecology and physiology of these gorgonians *in situ*, since results suggest that autotrophy can supply most of the metabolic needs in summer, while heterotrophy can compensate for the lack of autotrophic input in winter. However, more studies, in which seasonal changes in light level are coupled with changes in seawater temperature, are needed to complement these first data. Also, the response of *E. singularis* to a thermal stress seems independent of the trophic conditions. Indeed, conversely to some other cnidarian species, heterotrophy could not prevent bleaching under thermal stress, nor compensate for the decrease in the rates of photosynthesis, which induced, on a long-term, the decrease in the lipid reserves. By decreasing the rates of respiration (i.e. the energetic expenses) *E. singularis* was however able to resist the stress for some weeks, without necrosis, as already observed in previous laboratory experiments [15]–[17]. This is contrary to other gorgonian species such as *P.clavata* and *C.rubrum* [17]. In the light of these results, the mortality events of *E. singularis* observed in nature [63], [4], after seawater temperatures had reached values above 24°C, are either due to a long-term impairment of the photosynthesis, or to a combination of several stresses, such as a combination of temperature increase with variation in hydrodynamic conditions, UV radiation increases or presence of pathogens or contaminants.
